# Risk Factors for Unfavorable Pathological Types of Intravesical Recurrence in Patients With Upper Urinary Tract Urothelial Carcinoma Following Radical Nephroureterectomy

**DOI:** 10.3389/fonc.2022.834692

**Published:** 2022-04-13

**Authors:** Jun Zhu, Xiaoqing Zhang, Wei Yu, Xuesong Li, Zhisong He, Liqun Zhou, Zhongyuan Zhang, Gengyan Xiong

**Affiliations:** Department of Urology, Peking University First Hospital, Beijing, China

**Keywords:** upper urinary tract, urothelial carcinoma, nephroureterectomy, bladder recurrence, risk factors

## Abstract

**Background:**

Numerous studies have investigated the risk factors of intravesical recurrence (IVR) after radical nephroureterectomy (RNU) in patients with upper urinary tract urothelial carcinoma (UTUC). However, few studies explore the predictors for unfavorable pathological types of IVR following RNU.

**Methods:**

We retrospectively reviewed 155 patients diagnosed with bladder cancer (BC) following RNU. Binary logistic regression was used for the univariable and multivariable analyses. Nomograms were developed based on the multivariable analysis. The concordance index (C-index) was used to assess the performance of the nomograms. We performed internal validation by generating calibration plots.

**Results:**

Muscle-invasive BC (MIBC) was significantly correlated with operation interval (p = 0.004) and UTUC T-stage (p = 0.016). Operation interval (p = 0.002) and UTUC T-stage (p = 0.028) were also risk factors for BC > 3 cm. UTUC grade (p = 0.015), operation interval (p = 0.003), and hydronephrosis (p = 0.049) were independent predictors for high-grade BC (HGBC). MIBC (p = 0.018) and surgical approach (p = 0.003) were associated with multifocal IVR. Besides, MIBC and HGBC were associated with UTUC grade (p = 0.009), operation interval (p = 0.001), and hydronephrosis (p = 0.023). Moreover, only operation interval (p = 0.036) was a predictor for BC with at least one unfavorable pathological type. We developed nomograms for MIBC, HGBC, BC > 3 cm, and MIBC and/or HGBC. The calibration curves of the nomograms showed good agreement between the observation and prediction cases. The C-indexes of the nomograms were 0.820 (95% CI, 0.747–0.894), 0.728 (95% CI, 0.649–0.809), 0.770 (95% CI, 0.679–0.861), and 0.749 (95% CI, 0.671–0.827), respectively.

**Conclusions:**

The current study found that operation interval, UTUC T-stage, UTUC grade, surgical approach, and hydronephrosis are independent predictors for unfavorable pathological types of IVR following RNU. Nomograms based on these predictors were developed and internally validated to assess the risk of developing unfavorable pathological types of IVR. Furthermore, patients at high risk of developing unfavorable pathological types BC may benefit from more active follow-up 1 year after RNU by early detection of IVR.

## Introduction

Upper urinary tract urothelial carcinoma (UTUC) is a relatively uncommon disease and accounts for 5%–10% of urothelial carcinomas (1). About 60% of UTUCs are invasive at primary diagnosis. Radical nephroureterectomy (RNU) with the removal of bladder cuff excision is the gold standard treatment. Approximately 22%–47% of UTUC patients develop an intravesical recurrence (IVR) after RNU (2–4). Therefore, IVR monitoring following RNU is of great importance.

Although a few studies have looked into the risk factors for IVR after RNU for UTUC, few have tried to look into predictors for the malignant degree of secondary bladder cancer (BC) (5–7). Presently, IVR after RNU is managed according to the treatment guidelines for primary BC. Transurethral resection of the bladder tumor is recommended for favorable pathological types of IVR, while radical cystectomy (RC) is a curative treatment for most unfavorable pathological types of IVR (8, 9). Considering that advanced IVR after RNU predicts a worse prognosis and necessitates more aggressive treatment, it is critical to find predictors for unfavorable pathological types BC after RNU, such as muscle-invasive, high-grade, >3-cm-diameter, and multifocal IVR.

This study aimed to identify the independent risk factors and develop prediction models based on the predictors for unfavorable pathological types of IVR after RNU.

## Materials and Methods

### Patients

Our institutional review board approved this study. A review of the archived medical records of our hospital identified 169 patients who had IVR after undergoing RNU with curative intent for primary UTUC between January 2002 and September 2021 in Peaking University First Hospital, Beijing, China. We excluded three patients who had previous bladder tumors, two patients with a history of renal transplantation, and nine patients with incomplete follow-up data. Thus, this study included a total of 155 patients. Routine postoperative follow-up and cystoscopy were performed, and no bladder instillation treatment and adjuvant therapy were performed after RNU. Urologic ultrasound, CT, MRI, and ureteroscopy with or without biopsy were used to diagnose all the cases. Before surgery, cystoscopy and chest X-ray were performed to exclude concomitant bladder tumor and metastasis. Further, surgical methods include open and laparoscopic RNU. The distal ureter and bladder cuff excision was performed using the open extravesical technique and the intramural portion within the bladder wall through an open Gibson incision. Postoperative bladder instillation was not adapted after surgery.

### Clinical Variables and Tumor-Related Variables

The collected clinical data included age, sex, body mass index (BMI), smoking and drinking status, history of taking aristolochic acid (AA), preoperative ureteroscopic biopsy, hydronephrosis, surgical approach, neutrophil–lymphocyte ratio (NLR), operation interval (time between RNU and management of BC), and pathological features (multifocality, location, stage, grade, and maximum diameter). All tumors were staged by the Union for International Cancer Control TNM classification of malignant tumors in 2002 and graded according to the WHO classification of 2004. Besides, patients were grouped based on muscle-invasive BC (MIBC) and/or high-grade BC (HGBC) after RNU since patients must receive cystectomy based on the guideline. We also grouped patients by BC with at least one of the above four unfavorable pathological types of IVR to identify predictors for unfavorable pathological types of BC following RNU without dissecting around the kidney. The definition of smoking status was not homogeneous in different studies (10). Our study divided smoking status into three groups, non-smoker, former smoker, and current smoker, based on our data. All medical records were reviewed by two researchers independently.

### Statistical Analysis

All analyses were performed using R version 3.6.1 (The R Foundation) and SPSS (version 24.0, IBM, Armonk, NY, USA). Univariate binary logistic regression was performed for each variable to explore the independent predictors for unfavorable pathological types of IVR. Factors with a p-value <0.2 were included for stepwise multivariate binary logistic analysis. The calculation was done based on odds ratios (ORs) and 95% CI, and a value of p < 0.05 was considered statistically significant. Based on the multivariate binary logistic regression analysis results, nomograms were developed using R’s rms package version 3.0. The performance of the prediction model was measured using the concordance index (C-index). We conducted internal validation by constructing calibration plots and validated them with 500 bootstrap samples to reduce bias.

## Results

### Patients and Tumor Characteristics

**Table 1** summarizes the demographic information of included patients. The median patient age was 66 years (range: 31–87 years), and the median operation interval was 25.8 months (interquartile range (IQR), 7–33.5 months). Within 1 year of the primary procedure, 67 patients out of 155 (43.2%) were diagnosed with BC.

**Table 1:** Clinicopathologic characteristics of patients.

Characteristics	Number of patients No. (%) (n = 154)
Median age	68 (range, 31–87)
Mean operation interval	25.8 (IQR, 7–33.5 months)
Operation interval ≤12 months	67 (43.2)
Operation interval >12 months	88 (56.8)
Gender	
Male	80 (51.6)
Female	75 (48.4)
Median BMI	24.2 (IQR, 21.9–26.7)
BMI	
<25	96 (61.9)
≥25	59 (38.1)
Stage of UTUC	
<T2	64 (41.3)
≥T2	91 (58.7)
Grade of UTUC	
Low grade	49 (31.6)
High grade	106 (68.4)
Multifocal tumor	
Yes	33 (21.3)
No	122 (78.7)
Diameter of UTUC	
≤3 cm	97 (62.6)
>3 cm	58 (37.4)
Location	
Renal pelvis	68 (43.8)
Ureter	77 (49.7)
Both	10 (6.5)
Preoperative ureteroscopic biopsy	
Yes	25 (16.1)
No	130 (83.9)
Hydronephrosis	
Yes	92 (59.4)
No	63 (40.6)
AA	
Yes	10 (6.5)
No	145 (93.5)
Smoking	
Never	121 (78.1)
Former	9 (5.8)
Current	25 (16.1)
Drinking	
Yes	10 (6.5)
No	142 (93.5)
Surgical approach	
Open	59 (38.1)
Laparoscopic	96 (61.9)
Median NLR	2.6 (IQR, 1.9–3.9)
NLR ≥ 2.5	
Yes	82 (52.9)
No	73 (47.1)
MIBC	
Yes	16 (10.3)
No	139 (89.7)
HGBC	
Yes	69 (44.5)
No	86 (55.5)
Multifocal BC	
Yes	97 (62.6)
No	58 (37.4)
Diameter of BC	
≤3 cm	136 (87.7)
>3 cm	19 (12.3)
MIBC and/or HGBC	
Yes	71 (45.8)
No	84 (54.2)
BC with at least one unfavorable pathological type	
Yes	120 (77.4)
No	35 (22.6)

IQR, interquartile range; BMI, body mass index; UTUC, upper urinary tract urothelial carcinoma; NLR, neutrophil–lymphocyte ratio; MIBC, muscle-invasive bladder cancer; HGBC, high-grade bladder cancer; BC, bladder cancer.

### Independent Risk Factors for Unfavorable Pathological Types of Intravesical Recurrence

**Tables 2**, **3**, **4**, **5**, **6**, **7** present the results of univariate and multivariate analyses. For multivariate analysis, risk factors with a p-value of less than 0.2 were included. The operation interval (p = 0.004) and stage of primary UTUC (p = 0.016) were significantly correlated with MIBC after RNU. BC > 3 cm after RNU was also associated with operation interval (p = 0.002) and stage of primary UTUC (p = 0.028). High-grade IVR was predicted independently by UTUC grade (p = 0.015), operation interval (p = 0.003) and hydronephrosis (p = 0.049). MIBC (p = 0.018) and surgical approach (p = 0.003) were associated with multifocal IVR. Besides, following RNU, MIBC and HG-BC were linked to primary tumor grade (p = 0.009), operation interval (p = 0.001), and hydronephrosis (p = 0.023). Furthermore, only the operation interval (p = 0.036) was a predictor of BC with at least one unfavorable pathological type. Finally, we divided our research group into two groups based on the time between operations, with a 1-year cutoff. Operation interval was associated with all unfavorable pathological types of IVR except for the multifocal type in the more than 1 year group, while it was only correlated with MIBC in the other group (**Supplementary Tables 1–6**).

**Table 2:** Univariate and multivariate analyses for factors associated with MIBC after RNU.

	Univariate analysis		Multivariate analysis	
	OR (95% CI)	p-Value	OR (95% CI)	p-Value
Operation Interval	1.017 (1.003–1.032)	0.016	1.027 (1.009–1.450)	0.004
Stage of UTUC (<T2 *vs.*≥T2)	5.636 (1.234–25.742)	0.026	8.992 (1.499–53.108)	0.016
AA	0.963 (0.114–8.136)	0.972		
Age	1.038 (0.983–1.097)	0.180		
BMI	1.104 (0.95–1.284)	0.197		
BMI (<25 *vs.*≥25)	2.289 (0.803–6.518)	0.121	2.679 (0.855–8.401)	0.091
Diameter of UTUC (≤3 cm *vs.*>3* cm*)	0.525 (0.161–1.711)	0.285		
Drinking	0.706 (0.086–5.814)	0.746		
Gender	0.703 (0.248–1.994)	0.508		
Grade of UTUC	3.576 (0.780–16.395)	0.101		
Hydronephrosis	3.291 (0.897–12.07)	0.072	3.710 (0.899–15.311)	0.070
Multifocal tumor	0.223 (0.028–1.753)	0.154		
Surgical approach	1.027 (0.353–2.990)	0.961		
NLR	1.079 (0.980–1.188)	0.121		
NLR (<2.5 *vs.*≥2.5)	0.878 (0.312–2.473)	0.806		
Smoking	1.161 (0.350–3.856)	0.807		
Preoperative ureteroscopic biopsy	0.720 (0.153–3.387)	0.678		
Location		0.272		
(Ureter *vs.*renal pelvis)	2.667 (0.807–8.809)	0.108		
(Both *vs.*renal pelvis)	1.778 (0.178–17.727)	0.624		

MIBC, muscle-invasive bladder cancer; RNU, radical nephroureterectomy; OR, odds ratio; UTUC, upper urinary tract urothelial carcinoma; AA, aristolochic acid; BMI, body mass index; NLR, neutrophil–lymphocyte ratio.

**Table 3:** Univariate and multivariate analyses for factors associated with HGBC after RNU.

	Univariate analysis		Multivariate analysis	
	OR (95% CI)	p-Value	OR (95% CI)	p-Value
Operation Interval	1.023 (1.009–1.038)	0.034	1.022 (1.008–1.037)	0.003
Stage of UTUC (<T2 *vs.*≥T2)	2.039 (1.054–3.944)	0.316		
AA	1.952 (0.528–7.215)	0.253		
Age	1.018 (0.987–1.050)	0.826		
BMI	1.011 (0.921–1.109)	0.564		
BMI (<25 *vs.*≥25)	1.212 (0.631–2.325)	0.952		
Diameter of UTUC (≤3 cm *vs.*>3* cm*)	1.020 (0.530–1.963)	0.304		
Drinking	0.526 (0.155–1.789)	0.602		
Gender	0.845 (0.448–1.593)	0.008		
Grade of UTUC	2.696 (1.302–5.581)	0.098	2.636 (1.206–5.761)	0.015
Hydronephrosis	1.739 (0.903–3.351)	0.034	2.079 (1.005–4.303)	0.049
Multifocal tumor	0.766 (0.350–1.678)	0.505		
Surgical approach	0.422 (0.217–0.819)	0.011	0.511 (0.246–1.059)	0.071
NLR	1.062 (0.966–1.168)	0.212		
NLR (<2.5 *vs.*≥2.5)	0.769 (0.407–1.452)	0.418		
Smoking	1.433 (0.673–3.049)	0.351		
Preoperative ureteroscopic biopsy	0.532 (0.215–1.32)	0.174		
Location		0.163		
(Ureter *vs.*renal pelvis)	0.302 (1.418–0.731)	0.302		
(Both *vs.*renal pelvis)	3.769 (0.895–15.88)	0.071		

HGBC, high-grade bladder cancer; RNU, radical nephroureterectomy; OR, odds ratio; UTUC, upper urinary tract urothelial carcinoma; AA, aristolochic acid; BMI, body mass index; NLR, neutrophil–lymphocyte ratio.

**Table 4:** Univariate and multivariate analyses for factors associated with multifocal BC after RNU.

	Univariate analysis		Multivariate analysis	
	OR (95% CI)	p-Value	OR (95% CI)	p-Value
Operation Interval	1.009 (0.996–1.022)	0.167		
Stage of UTUC (<T2 *vs.*≥T2)	0.499 (0.252–0.989)	0.046	0.416 (0.201–0.860)	0.018
AA	2.517 (0.516–12.282)	0.254		
Age	0.981 (0.950–1.012)	0.227		
BMI	0.965 (0.877–1.061)	0.459		
BMI (<25 *vs.*≥25)	1.135 (0.579–2.224)	0.713		
Diameter of UTUC (≤3 cm *vs.*>3* cm*)	1.224 (0.621–2.410)	0.559		
Drinking	0.674 (0.215–2.113)	0.499		
Gender	1.238 (0.645–2.375)	0.521		
Grade of UTUC	1.088 (0.542–2.185)	0.813		
Hydronephrosis	0.520 (0.262–1.031)	0.061	0.593 (0.288–1.220)	0.155
Multifocal tumor	1.059 (0.477–2.353)	0.888		
Surgical approach	0.368 (0.179–0.757)	0.007	0.322 (0.151–0.686)	0.003
NLR	0.979 (0.901–1.065)	0.624		
NLR (<2.5 *vs.*≥2.5)	0.551 (0.284–1.070)	0.078	0.560 (0.280–1.121)	0.102
Smoking	0.868 (0.401–1.878)	0.720		
Preoperative ureteroscopic biopsy	1.328 (0.534–3.305)	0.542		
Location		0.379		
(Ureter *vs.*renal pelvis)	0.638 (0.323–1.258)	0.194		
(Both *vs.*renal pelvis)	1.116 (0.263–4.733)	0.882		

BC, bladder cancer; RNU, radical nephroureterectomy; OR, odds ratio; UTUC, upper urinary tract urothelial carcinoma; AA, aristolochic acid; BMI, body mass index; NLR, neutrophil–lymphocyte ratio.

**Table 5:** Univariate and multivariate analyses for factors associated with BC greater than 3 cm after RNU.

	Univariate analysis		Multivariate analysis	
	OR (95% CI)	p-Value	OR (95% CI)	p-Value
Operation Interval	1.019 (1.006–1.033)	0.005	1.024 (1.009–1.039)	0.002
Stage of UTUC (<T2 *vs.*≥T2)	2.961 (0.934–9.385)	0.065	4.286 (1.166–15.756)	0.028
Age	1.002 (0.957–1.049)	0.922		
BMI	1.101 (0.957–1.266)	0.180		
BMI (<25 *vs.*≥25)	1.973 (0.751–5.184)	0.168		
Diameter of UTUC (≤3 cm *vs.*>3* cm*)	1.251 (0.472–3.317)	0.653		
Drinking	0.574 (0.070–4.683)	0.604		
Gender	1.048 (0.401–2.740)	0.924		
Grade of UTUC	1.854 (0.582–5.911)	0.296		
Hydronephrosis	1.200 (0.445–3.238)	0.719		
Multifocal tumor	1.258 (0.704–2.249)	0.534		
Surgical approach	0.646 (0.246–1.697)	0.375		
NLR	0.663 (0.181–2.426)	0.196		
NLR (<2.5 *vs.*≥2.5)	1.064 (0.968–1.170)	0.607		
Smoking	0.777 (0.297–2.032)	0.678		
Preoperative ureteroscopic biopsy	1.262 (0.421–3.786)	0.483		

BC, bladder cancer; RNU, radical nephroureterectomy; OR, odds ratio; UTUC, upper urinary tract urothelial carcinoma; AA, aristolochic acid; BMI, body mass index; NLR, neutrophil–lymphocyte ratio.

**Table 6:** Univariate and multivariate analyses for factors associated with MIBC and/or HGBC after RNU.

	Univariate analysis		Multivariate analysis	
	OR (95% CI)	p-Value	OR (95% CI)	p-Value
Operation Interval	1.026 (1.011–1.042)	0.001	1.025 (1.010–1.041)	0.001
Stage of UTUC (<T2 *vs.*≥T2)	2.227 (1.151–4.310)	0.017		
AA	1.846 (0.500–6.820)	0.358		
Age	1.024 (0.993–1.056)	0.134		
BMI	0.995 (0.906–1.091)	0.907		
BMI (<25 *vs.*≥25)	1.113 (0.581–2.133)	0.746		
Diameter of UTUC (≤3 cm *vs.*>3* cm*)	1.172 (0.610–2.251)	0.633		
Drinking	0.498 (0.146–1.690)	0.263		
Gender	0.759 (0.403–1.430)	0.394		
Grade of UTUC	2.908 (1.404–6.022)	0.004	2.889 (1.307–6.386)	0.009
Hydronephrosis	1.897 (0.984–3.656)	0.056	2.360 (1.125–4.953)	0.023
Multifocal tumor	0.717 (0.328–1.570)	0.406		
Surgical approach	0.411 (0.212–0.799)	0.009	0.496 (0.236–1.042)	0.064
NLR	1.057 (0.962–1.161)	0.248		
NLR (<2.5 *vs.*≥2.5)	0.765 (0.406–1.443)	0.408		
Smoking	1.339 (0.629–2.846)	0.449		
Preoperative ureteroscopic biopsy	2.462 (1.247–4.859)	0.009		
Location		0.189		
(Ureter *vs.*renal pelvis)	1.405 (0.726–2.718)	0.313		
(Both *vs.*renal pelvis)	3.543 (0.842–14.911)	0.084		

MIBC, muscle-invasive bladder cancer; HGBC, high-grade bladder cancer; RNU, radical nephroureterectomy; OR, odd ratio; UTUC, upper urinary tract urothelial carcinoma; AA, aristolochic acid; BMI, body mass index; NLR, neutrophil–lymphocyte ratio.

**Table 7:** Univariate and multivariate analyses for factors associated with BC with at least one unfavorable pathological type after RNU.

	Univariate analysis		Multivariate analysis	
	OR (95% CI)	p-Value	OR (95% CI)	p-Value
Operation Interval	1.031 (1.006–1.058)	0.016	1.027 (1.002–1.053)	0.036
Stage of UTUC (<T2 *vs.*≥T2)	0.933 (0.433–2.011)	0.860		
AA	2.757 (0.337–22.544)	0.344		
Age	0.999 (0.963–1.035)	0.940		
BMI	1.016 (0.909–1.135)	0.777		
BMI (<25 *vs.*≥25)	1.725 (0.761–3.912)	0.192		
Diameter of UTUC (≤3 cm *vs.*>3* cm*)	1.015 (0.466–2.213)	0.969		
Drinking	0.429 (0.131–1.406)	0.162		
Gender	0.871 (0.409–1.853)	0.719		
Grade of UTUC	1.619 (0.740–3.541)	0.227		
Hydronephrosis	0.966 (0.448–2.082)	0.930		
Multifocal tumor	0.722 (0.300–1.741)	0.469		
Surgical approach	0.327 (0.132–0.807)	0.015	0.412 (0.163–1.042)	0.061
NLR	1.029 (0.916–1.156)	0.633		
NLR (<2.5 *vs.*≥2.5)	0.591 (0.273–1.281)	0.182		
Smoking	0.799 (0.334–1.914)	0.615		
Preoperative ureteroscopic biopsy	0.909 (0.332–2.488)	0.853		
Location		0.959		
(Ureter *vs.*renal pelvis)	1.086 (0.499–2.362)	0.835		
(Both *vs.*renal pelvis)	1.231 (0.237–6.394)	0.805		

BC, bladder cancer; RNU, radical nephroureterectomy; OR, odds ratio; UTUC, upper urinary tract urothelial carcinoma; AA, aristolochic acid; BMI, body mass index; NLR, neutrophil–lymphocyte ratio.

### Prediction Models for Unfavorable Pathological Types of Intravesical Recurrence

**Figure 1:**
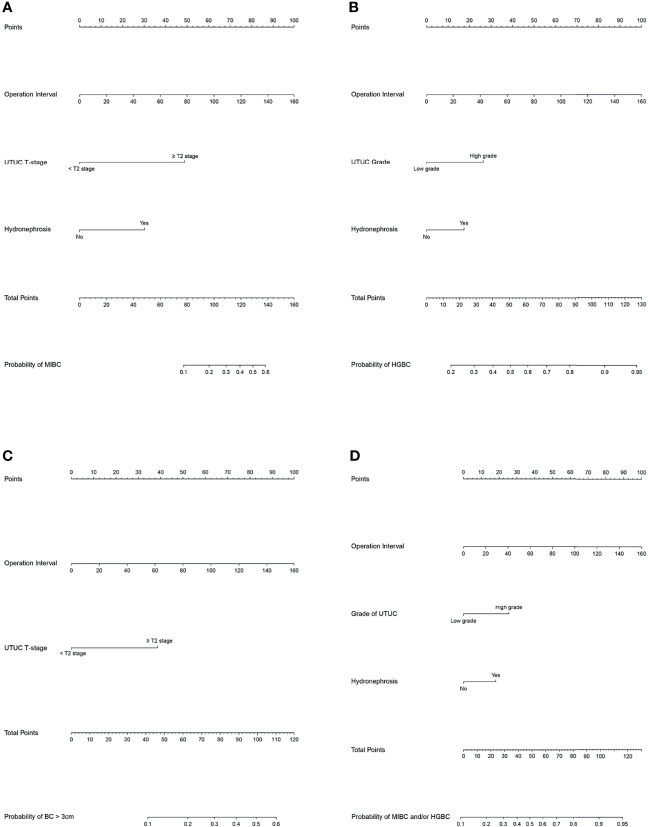
Nomograms for the prediction of unfavorable pathological types intravesical recurrence (IVR) following radical nephroureterectomy (RNU). **(A)** Nomogram to predict the risk of muscle-invasive bladder cancer (MIBC) after RNU. **(A)** Bomogram to predict the risk of high-grade bladder cancer (HGBC) after RNU. **(C)** Nomogram to predict the risk of bladder cancer >3 cm after RNU. **(D)** Nomogram to predict the risk of MIBC and/or HGBC after RNU. To use the nomogram, first, assign the points of each variable of the patient by drawing a line straight up to the top line labeled “Points”. Sum up the points of the risk factors as total points. Then draw a vertical line down from the axis labeled “Total Points” to the bottom line to get the predicted possibility of unfavorable pathological types intravesical recurrence (IVR) following RNU.

**Figure 2:**
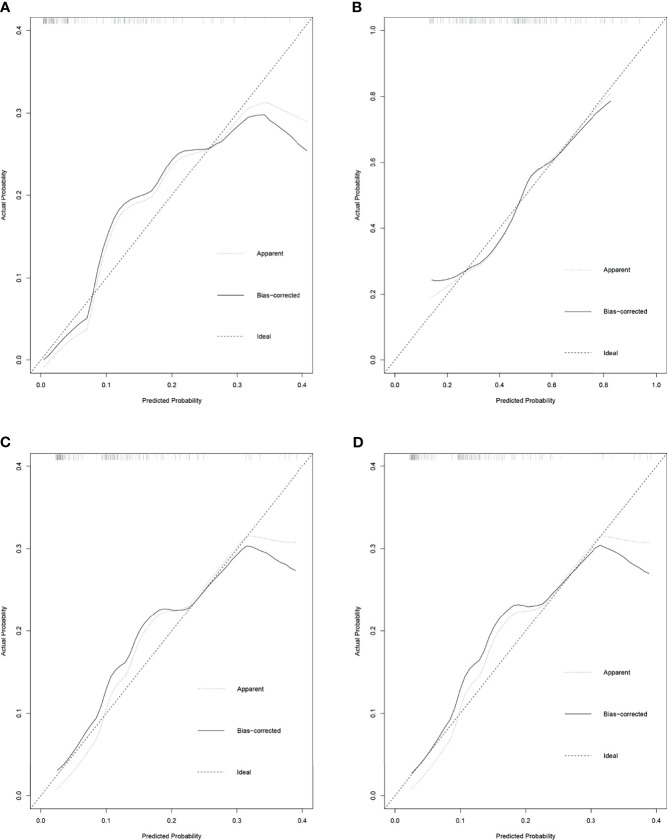
The calibration curves for the prediction nomograms of unfavorable pathological types intravesical recurrence following radical nephroureterectomy (RNU). **(A)** Calibration curves for the prediction nomograms of muscle-invasive bladder cancer (MIBC) after RNU. **(B)** Calibration curves for the prediction nomograms of high-grade bladder cancer (HGBC) after RNU. **(C)** Calibration curves for the prediction nomograms of bladder cancer >3 cm after RNU. **(D)** Calibration curves for the prediction nomograms of MIBC and/or HGBC after RNU.

These independent risk factors were pooled to develop the prediction models. We developed nomograms for MIBC, HGBC, BC > 3 cm, and MIBC and/or HGBC. The nomograms are presented in **Figure 1**. The calibration curves of the prediction models demonstrated good agreement between the observation and prediction cases in our cohort (**Figure 2**). Moreover, the C-indexes of the above nomograms were 0.820 (95% CI, 0.747–0.894), 0.728 (95% CI, 0.649–0.809), 0.770 (95% CI, 0.679–0.861), and 0.749 (95% CI, 0.671–0.827).

## Discussion

UTUC is a relatively rare malignancy, accounting for 5%–10% of urothelial carcinomas. Despite significant advances in diagnosis and management, 22%–47% of patients still develop IVR after RNU (3, 11). Many studies have identified risk factors for IVR following RNU, including T-stage, multifocality, diagnostic preoperative ureteroscopic biopsy, male sex, previous BC, smoking, chronic kidney disease, necrosis, tumor location, transurethral resection of the bladder cuff, positive resection margin, and surgical approach (7, 12–16). However, limited data are available about unfavorable pathological types of BC that develop after RNU. Our study explores the predictors and establishes prediction models for unfavorable pathological types of IVR following RNU.

The present study showed that operation interval and T-stage of primary UTUC were risk factors for MIBC and BC > 3 cm after RNU, consistent with Kim’s results (17). We first found that grade of UTUC (p = 0.015), hydronephrosis (p = 0.049), and operation interval (p = 0.003) were independent risk factors for HGBC. Besides, MIBC and HGBC following RNU were associated with UTUC grade (p = 0.009), operation interval (p = 0.001), and hydronephrosis (p = 0.023). These findings support the intraluminal seeding hypothesis as a predominant mechanism of IVR following RNU (7, 18). Moreover, MIBC (p = 0.018) and surgical approach (p = 0.003) were associated with multifocal BC after RNU. Open surgery is a risk factor for multifocal IVR, which may cause a poor prognosis, consistent with a randomized prospective study (15). Only operation interval (p = 0.036) was a predictor for BC with at least one unfavorable pathological type, suggesting that follow-up after RNU is of great importance. Smoking at the time of RC is significantly correlated with increased risk for postoperative infections, complications, and perioperative mortality.

We chose the time between two procedures as an indicator to explore the relationship between time after RNU and the malignant degree of IVR, considering it is an objective variable. Operation interval was a predictor for all unfavorable pathological types of IVR except multifocal type. We further investigated the influence of operation interval on the malignant degree of IVR by dividing the cohort into two groups with a cutoff of 1 year. Operation interval was correlated with MIBC, HGBC, BC > 3 cm, and MIBC and/or HGBC in the more than 1 year group. However, operation interval was only associated with MIBC in the other group. There are currently different opinions on an optimal surveillance regimen in UTUC patients after RNU (19–21). It is widely accepted that patients with low-risk tumors are recommended to receive cystoscopy at 3 months. If negative, perform cystoscopy after 9 months and then yearly for 5 years. For patients with high-risk tumors, both cystoscopy and urinary cytology should be performed at 3 months. If negative, perform repeat cystoscopy and cytology every 3 months for 2 years, every 6 months after that until 5 years, and then yearly. Further, CT urography and chest CT should be performed every 6 months for 2 years, and then once a year. Most studies took 2 years as a cutoff when exploring the follow-up protocol (4, 22–25). However, operation interval was an independent risk factor for unfavorable pathological types of IVR except for the multifocal type in the more than 1 year group, while only associated with MIBC in the other group. Moreover, previous studies have shown that the risk of recurrence and death increases during the follow-up (26). Hence, patients with a high risk of developing unfavorable pathological types BC may benefit from more active follow-up 1 year after RNU by early detection of recurrence. We also established prediction models for unfavorable pathological types BC, which can help identify patients with a high risk of developing unfavorable pathological types of IVR.

Notably, the present study has several limitations because it was a retrospective study that may have caused inherent selection bias and was conducted on a single hospital. Besides, the sample size was relatively small. Moreover, the predictive models were internally validated to exhibit good performance; thus, external validation by long-term follow-up in multiple centers is still required to verify the utility of the nomograms.

## Conclusion

To summarize, the current study identified independent risk factors for unfavorable pathological types of BC developing after RNU, such as operation interval, UTUC T-stage, UTUC grade, surgical approach, and hydronephrosis. To assess the risk of developing unfavorable pathological types BC after RNU, accurate prediction models based on these predictors were developed and internally validated. Furthermore, patients at high risk of developing unfavorable pathological types BC may benefit from more active follow-up 1 year after RNU by early diagnosis of IVR.

## Data Availability Statement

The raw data supporting the conclusions of this article will be made available by the authors, without undue reservation.

## Ethics Statement

The studies involving human participants were reviewed and approved by Peking University First Hospital Institutional Review Board. Written informed consent for participation was not required for this study in accordance with the national legislation and the institutional requirements.

## Author Contributions

GX and ZZ conceived the study. JZ and XZ extracted all the data and performed the analyses. LZ, ZH, XL, and WY supervised the project and provided guidance throughout the preparation of this manuscript. JZ and XZ prepared the tables and figures. JZ wrote the manuscript. GX provided funding. All authors contributed to and revised the final manuscript.

## Funding

This work was supported by funds from the Capital’s Funds for Health Improvement and Research (2020–4–40712) and the National Natural Science Foundation of China (81902871).

## Conflict of Interest

The authors declare that the research was conducted in the absence of any commercial or financial relationships that could be construed as a potential conflict of interest.

## Publisher’s Note

All claims expressed in this article are solely those of the authors and do not necessarily represent those of their affiliated organizations, or those of the publisher, the editors and the reviewers. Any product that may be evaluated in this article, or claim that may be made by its manufacturer, is not guaranteed or endorsed by the publisher.

## Abbreviations

UTUC, upper urinary tract urothelial carcinoma; RNU, radical nephroureterectomy; IVR, intravesical recurrence; BC, bladder cancer; BMI, body mass index; AA, aristolochic acid; NLR, neutrophil–lymphocyte ratio; MIBC, muscle-invasive bladder cancer; HGBC, high-grade bladder cancer; OR, odds ratio; C-index, concordance index; IQR, interquartile range.
