# Supplementary material for: Risk Factors for Unfavorable Pathological Types of Intravesical Recurrence in Patients With Upper Urinary Tract Urothelial Carcinoma Following Radical Nephroureterectomy
Source: Front Oncol. 2022 Apr 13;12:834692. doi: 10.3389/fonc.2022.834692 (PMC9043951; doi:10.3389/fonc.2022.834692)
Supplement: Supplementary file 1 [file DataSheet_1.docx]

**Supplementary Table 1** Univariate and multivariate analyses for factors associated with MIBC after RNU

|  | **Operation interval** $\boldsymbol{\leq}$ **1 year** | | | | **Operation interval** $\boldsymbol{>}$ **1 year** | | | |
| --- | --- | --- | --- | --- | --- | --- | --- | --- |
|  | **Univariate analysis** | | **Multivariate analysis** | | **Univariate analysis** | | **Multivariate analysis** | |
|  | OR (95% CI) | P | OR (95% CI) | P | OR (95% CI) | P | OR (95% CI) | P |
| Operation Interval | 1.526(1.017-2.290) | 0.041 | 1.874(1.078-3.257) | 0.026 | 1.018(1.000-1.035) | 0.045 | 1.024(1.002-1.046) | 0.028 |
| Stage of UTUC ($<$T2 vs. $\geq$T2) |  |  |  |  | 3.750(0.760-18.509) | 0.105 | 7.483(1.054-53.137) | 0.044 |
| AA |  |  |  |  | 1.183(0.129-0.875) | 0.882 |  |  |
| Age | 0.974(0.898-1.056) | 0.524 |  |  | 1.081(1.001-1.168) | 0.048 |  |  |
| BMI | 1.056(0.800-1.394) | 0.701 |  |  | 1.121(0.938-1.339) | 0.210 |  |  |
| BMI ($<$25 vs. $\geq$25) | 2.727(0.423-17.577) | 0.291 |  |  | 2.100(0.587-7.511) | 0.254 |  |  |
| Diameter of UTUC  ($\leq$ 3cm vs. $>$ 3cm) | 0.455(0.048-4.322) | 0.493 |  |  | 0.527(0.130-2.143) | 0.371 |  |  |
| Drinking | 1.964(0.191-20.152) | 0.570 |  |  |  |  |  |  |
| Gender |  |  |  |  | 0.195(0.040-0.963) | 0.045 | 0.174(0.033-0.922) | 0.040 |
| Grade of UTUC |  |  |  |  | 2.163(0.435-10.766) | 0.346 |  |  |
| Hydronephrosis |  |  |  |  | 1.896(0.467-7.707) | 0.371 |  |  |
| Multifocal tumor | 0.783(0.081-7.560) | 0.833 |  |  |  |  |  |  |
| Surgical approach |  |  |  |  | 0.732(0.206-2.601) | 0.629 |  |  |
| NLR | 1.067(0.714-1.594) | 0.754 |  |  | 1.073(0.971-1.185) | 0.167 |  |  |
| NLR ($<$2.5 vs. $\geq$2.5) | 0.625(0.098-4.004) | 0.620 |  |  | 1.000(0.281-3.556) | 1.000 |  |  |
| Smoking | 5.143(0.780-33.894) | 0.089 |  |  | 0.353(0.042-2.955) | 0.337 |  |  |
| Ureteroscopy | 0.857(0.088-8.303) | 0.894 |  |  | 0.756(0.086-6.617) | 0.800 |  |  |

**Abbreviations:** MIBC, muscle-invasive bladder cancer; RNU, Radical nephroureterectomy; OR, odds ratio; CI=confidence interval; UTUC, upper urinary tract urothelial carcinoma; AA, aristolochic acid; BMI, body mass index; NLR, neutrophil-lymphocyte ratio.

**Supplementary Table 2** Univariate and multivariate analyses for factors associated with HGBC after RNU

|  | **Operation interval** $\boldsymbol{\leq}$ **1 year** | | | | **Operation interval** $\boldsymbol{>}$ **1 year** | | | |
| --- | --- | --- | --- | --- | --- | --- | --- | --- |
|  | **Univariate analysis** | | **Multivariate analysis** | | **Univariate analysis** | | **Multivariate analysis** | |
|  | OR (95% CI) | P | OR (95% CI) | P | OR (95% CI) | P | OR (95% CI) | P |
| Operation Interval | 1.017(0.846-1.223) | 0.858 |  |  | 1.019(1.001-1.036) | 0.035 | 1.019 (1.001-1.037) | 0.036 |
| Stage of UTUC ($<$T2 vs. $\geq$T2) | 2.933(0.921-9.347) | 0.069 |  |  | 1.824(0.774-4.295) | 0.169 |  |  |
| AA | 1.100(0.094-12.849) | 0.939 |  |  | 2.209(0.405-12.056) | 0.360 |  |  |
| Age | 1.019(0.968-1.073) | 0.466 |  |  | 1.017(0.977-1.059) | 0.404 |  |  |
| BMI | 0.904(0.769-1.064) | 0.224 |  |  | 1.074(0.950-1.214) | 0.255 |  |  |
| BMI ($<$25 vs. $\geq$25) | 0.778(0.263-2.298) | 0.649 |  |  | 1.615(0.674-3.869) | 0.282 |  |  |
| Diameter of UTUC  ($\leq$ 3cm vs. $>$ 3cm) | 0.682(0.223-2.092) | 0.504 |  |  | 1.190(0.504-2.812) | 0.691 |  |  |
| Drinking | 0.702(0.129-3.807) | 0.681 |  |  | 0.536(0.085-3.379) | 0.507 |  |  |
| Gender | 2.727(0.899-8.271) | 0.076 | 3.484(1.003-12.097) | 0.049 | 0.437(0.185-1.029) | 0.058 |  |  |
| Grade of UTUC | 4.222(1.088-16.383) | 0.037 | 5.371(1.306-22.094) | 0.020 | 2.242(0.890-5.648) | 0.087 |  |  |
| Hydronephrosis | 1.680(0.572-4.932) | 0.345 |  |  | 1.810(0.763-4.29) | 0.178 |  |  |
| Multifocal tumor | 0.667(0.187-2.380) | 0.532 |  |  | 0.923(0.320-2.667) | 0.882 |  |  |
| Surgical approach | 0.281(0.089-0.889) | 0.031 | 0.195(0.050-0.765) | 0.019 | 0.680(0.292-1.583) | 0.371 |  |  |
| NLR | 0.949(0.731-1.233) | 0.697 |  |  | 1.077(0.953-1.218) | 0.235 |  |  |
| NLR ($<$2.5 vs. $\geq$2.5) | 0.833(0.296-2.342) | 0.730 |  |  | 0.667(0.285-1.558) | 0.349 |  |  |
| Smoking | 2.530(0.807-7.934) | 0.111 |  |  | 1.053(0.371-2.984) | 0.923 |  |  |
| Ureteroscopy | 0.472(0.118-1.892) | 0.289 |  |  | 0.814(0.218-3.039) | 0.759 |  |  |
| Location |  | 0.220 |  |  |  | 0.520 |  |  |
| (ureter vs. renal pelvis) | 1.886 (0.616-5.768) | 0.266 |  |  | 1.128(0.474-2.685) | 0.786 |  |  |
| (both vs. renal pelvis) | 5.143 (0.712-37.151) | 0.105 |  |  | 3.789(0.386-37.203) | 0.253 |  |  |

**Abbreviations:** HGBC, high-grade bladder cancer; RNU, Radical nephroureterectomy; OR, odds ratio; CI=confidence interval; UTUC, upper urinary tract urothelial carcinoma; AA, aristolochic acid; BMI, body mass index; NLR, neutrophil-lymphocyte ratio.

**Supplementary Table 3** Univariate and multivariate analyses for factors associated with multifocal BC after RNU.

|  | | **Operation interval** $\boldsymbol{\leq}$ **1 year** | | | | **Operation interval** $\boldsymbol{>}$ **1 year** | | | |
| --- | --- | --- | --- | --- | --- | --- | --- | --- | --- |
|  |  | **Univariate analysis** | | **Multivariate analysis** | | **Univariate analysis** | | **Multivariate analysis** | |
|  | OR (95% CI) | | P | OR (95% CI) | P | OR (95% CI) | P | OR (95% CI) | P |
| Operation Interval | 1.009(0.849-1.199) | | 0.919 |  |  | 1.004(0.989-1.020) | 0.578 | 0.558(0.208-1.499) | 0.247 |
| Stage of UTUC ($<$T2 vs. $\geq$T2) | 0.588(0.217-1.594) | | 0.297 |  |  | 0.428(0.163-1.121) | 0.084 | 0.473(0.177-1.264) | 0.135 |
| AA |  | |  |  |  | 1.182(0.215-6.501) | 0.848 |  |  |
| Age | 0.949(0.900-1.000) | | 0.049 | 0.945(0.895-0.998) | 0.041 | 1.003(0.962-1.047) | 0.880 |  |  |
| BMI | 0.961(0.829-1.113) | | 0.595 |  |  | 0.963(0.847-1.095) | 0.565 |  |  |
| BMI ($<$25 vs. $\geq$25) | 1.778(0.643-4.912) | | 0.267 |  |  | 0.772(0.309-1.926) | 0.579 |  |  |
| Diameter of UTUC | 1.083(0.392-2.992) | | 0.877 |  |  | 1.286(0.508-3.252) | 0.596 |  |  |
| ($\leq$ 3cm vs. $>$ 3cm) |  | |  |  |  |  |  |  |  |
| Drinking | 0.788(0.180-3.455) | | 0.752 |  |  | 0.684(0.108-4.344) | 0.687 |  |  |
| Gender | 1.148(0.436-3.024) | | 0.779 |  |  | 1.425(0.577-3.518) | 0.442 |  |  |
| Grade of UTUC | 1.042(0.374-2.902) | | 0.938 |  |  | 1.105(0.421-2.905) | 0.839 |  |  |
| Hydronephrosis | 0.528(0.195-1.429) | | 0.208 |  |  | 0.489(0.186-1.283) | 0.146 |  |  |
| Multifocal tumor | 1.056(0.341-3.274) | | 0.925 |  |  | 1.150(0.362-3.652) | 0.813 |  |  |
| Surgical approach | 0.284(0.081-0.992) | | 0.049 | 0.256(0.070-0.938) | 0.040 | 0.473(0.177-1.264) | 0.135 |  |  |
| NLR | 0.970(0.768-1.224) | | 0.797 |  |  | 0.972(0.888-1.065) | 0.547 |  |  |
| NLR ($<$2.5 vs. $\geq$2.5) | 0.508(0.191-1.351) | | 0.175 |  |  | 0.556(0.221-1.400) | 0.212 |  |  |
| Smoking | 0.884(0.293-2.666) | | 0.827 |  |  | 0.917(0.304-2.760) | 0.877 |  |  |
| Ureteroscopy | 1.286(0.400-4.134) | | 0.673 |  |  | 2.000(0.396-10.100) | 0.402 |  |  |
| Location |  | | 0.245 |  |  |  | 0.695 |  |  |
| (ureter vs. renal pelvis) | 0.520 (0.189-1.430) | | 0.205 |  |  | 0.694(0.270-1.788) | 0.450 |  |  |
| (both vs. renal pelvis) | 2.526 (0.251-25.385) | | 0.431 |  |  | 0.566(0.080-3.830) | 0.551 |  |  |

**Abbreviations:** BC, bladder cancer; RNU, Radical nephroureterectomy; OR, odds ratio; CI=confidence interval; UTUC, upper urinary tract urothelial carcinoma; AA, aristolochic acid; BMI, body mass index; NLR, neutrophil-lymphocyte ratio;

**Supplementary Table 4** Univariate and multivariate analyses for factors associated with BC $>$ 3 cm after RNU.

|  | | **Operation interval** $\boldsymbol{\leq}$ **1 year** | | | | **Operation interval** $\boldsymbol{>}$ **1 year** | | | |
| --- | --- | --- | --- | --- | --- | --- | --- | --- | --- |
|  |  | **Univariate analysis** | | **Multivariate analysis** | | **Univariate analysis** | | **Multivariate analysis** | |
|  | OR (95% CI) | | P | OR (95% CI) | P | OR (95% CI) | P-value | OR (95% CI) | P |
| Operation Interval | 1.056(0.800-1.394) | | 0.701 | 1.699(0.972-2.968) | 0.063 | 1.018(1.001-1.034) | 0.034 | 1.018(1.001-1.034) | 0.034 |
| Stage of UTUC ($<$T2 vs. $\geq$T2) | 4.856(1.050-22.459) | | 0.043 |  |  | 2.012(0.578-7.001) | 0.272 |  |  |
| AA | 1.526(1.017-2.290) | | 0.041 |  |  |  |  |  |  |
| Age |  | |  |  |  | 1.013(0.959-1.071) | 0.640 |  |  |
| BMI | 5.143(0.780-33.894) | | 0.089 |  |  | 1.112(0.946-1.308) | 0.198 |  |  |
| BMI ($<$25 vs. $\geq$25) |  | |  |  |  | 1.741(0.551-5.495) | 0.345 |  |  |
| Diameter of UTUC  ($\leq$ 3cm vs. $>$ 3cm) | 2.727(0.423-17.577) | | 0.291 |  |  | 1.643(0.521-5.179) | 0.397 |  |  |
| Drinking | 1.067(0.714-1.594) | | 0.754 |  |  |  |  |  |  |
| Gender | 0.783(0.081-7.560) | | 0.833 |  |  | 0.526(0.161-1.720) | 0.288 |  |  |
| Grade of UTUC | 0.857(0.088-8.303) | | 0.894 |  |  | 1.127(0.320-3.974) | 0.852 |  |  |
| Hydronephrosis | 0.974(0.898-1.056) | | 0.524 |  |  | 0.609(0.193-1.919) | 0.397 |  |  |
| Multifocal tumor | 0.455(0.048-4.322) | | 0.493 |  |  | 0.656(0.132-3.249) | 0.605 |  |  |
| Surgical approach |  | |  |  |  | 0.447(0.137-1.463) | 0.183 |  |  |
| NLR | 0.625(0.098-4.004) | | 0.620 |  |  | 1.054(0.956-1.161) | 0.291 |  |  |
| NLR ($<$2.5 vs. $\geq$2.5) |  | |  |  |  | 0.805(0.257-2.526) | 0.710 |  |  |
| Smoking |  | |  |  |  | 0.604(0.122-2.980) | 0.536 |  |  |
| Ureteroscopy |  | |  |  |  | 0.556(0.065-4.770) | 0.592 |  |  |
| Location |  | |  |  |  |  | 0.960 |  |  |
| (ureter vs. renal pelvis) |  | |  |  |  | 0.927(0.283-3.042) | 0.901 |  |  |
| (both vs. renal pelvis) |  | |  |  |  | 1.292(0.122-13.670) | 0.832 |  |  |

**Abbreviations:** BC, bladder cancer; RNU, Radical nephroureterectomy; OR, odds ratio; CI=confidence interval; UTUC, upper urinary tract urothelial carcinoma; AA, aristolochic acid; BMI, body mass index; NLR, neutrophil-lymphocyte ratio.

**Supplementary Table 5** Univariate and multivariate analyses for factors associated with MIBC and/or HGBC after RNU.

|  | **Operation interval** $\boldsymbol{\leq}$ **1 year** | | | | **Operation interval** $\boldsymbol{>}$ **1 year** | | | |
| --- | --- | --- | --- | --- | --- | --- | --- | --- |
|  | **Univariate analysis** | | **Multivariate analysis** | | **Univariate analysis** | | **Multivariate analysis** | |
|  | OR (95% CI) | P | OR (95% CI) | P | OR (95% CI) | P | OR (95% CI) | P |
| Operation Interval | 1.017(0.846-1.223) | 0.858 |  |  | 1.021(1.002-1.039) | 0.027 | 1.022(1.004-1.041) | 0.018 |
| Stage of UTUC ($<$T2 vs. $\geq$T2) | 2.933(0.921-9.347) | 0.069 |  |  | 2.157(0.908-5.121) | 0.081 | 2.951(1.122-7.761) | 0.028 |
| AA | 1.100(0.094-12.849) | 0.939 |  |  | 2.000(0.366-10.919) | 0.423 |  |  |
| Age | 1.019(0.968-1.073) | 0.466 |  |  | 1.027(0.987-1.070) | 0.191 |  |  |
| BMI | 0.904(0.769-1.064) | 0.224 |  |  | 1.045(0.925-1.180) | 0.481 |  |  |
| BMI ($<$25 vs. $\geq$25) | 0.778(0.263-2.298) | 0.649 |  |  | 1.393(0.581-3.339) | 0.458 |  |  |
| Diameter of UTUC  ($\leq$ 3cm vs. $>$ 3cm) | 0.682(0.223-2.092) | 0.504 |  |  | 1.511(0.632-3.615) | 0.354 |  |  |
| Drinking | 0.702(0.129-3.807) | 0.681 |  |  | 0.486(0.077-3.065) | 0.443 |  |  |
| Gender | 2.727(0.899-8.271) | 0.076 | 3.484(1.003-12.097) | 0.049 | 0.358(0.149-0.855) | 0.021 | 0.289(0.110-0.759) | 0.012 |
| Grade of UTUC | 4.222(1.088-16.383) | 0.037 | 7.870(1.620-38.239) | 0.020 | 2.579(1.019-6.527) | 0.046 |  |  |
| Hydronephrosis | 1.680(0.572-4.932) | 0.345 |  |  | 2.125(0.890-5.074) | 0.090 |  |  |
| Multifocal tumor | 0.667(0.187-2.380) | 0.532 |  |  | 0.823(0.285-2.382) | 0.720 |  |  |
| Surgical approach | 0.281(0.089-0.889) | 0.031 | 0.195(0.050-0.765) | 0.019 | 0.671(0.287-1.571) | 0.358 |  |  |
| NLR | 0.949(0.731-1.233) | 0.697 |  |  | 1.068(0.947-1.204) | 0.281 |  |  |
| NLR ($<$2.5 vs. $\geq$2.5) | 0.833(0.296-2.342) | 0.730 |  |  | 0.652(0.277-1.533) | 0.327 |  |  |
| Smoking | 2.530(0.807-7.934) | 0.111 |  |  | 0.938(0.330-2.661) | 0.903 |  |  |
| Ureteroscopy | 0.472(0.118-1.892) | 0.289 |  |  | 0.733(0.196-2.741) | 0.645 |  |  |
| Location |  | 0.220 |  |  |  | 0.576 |  |  |
| (ureter vs. renal pelvis) | 1.886(0.616-5.768) | 0.266 |  |  | 1.105(0.463-2.639) | 0.822 |  |  |
| (both vs. renal pelvis) | 5.143(0.712-37.151) | 0.105 |  |  | 3.400(0.346-33.397) | 0.294 |  |  |

Abbreviations: MIBC, muscle-invasive bladder cancer; HGBC, high-grade bladder cancer; RNU, Radical nephroureterectomy; OR, odd ratio; CI=confidence interval; UTUC, upper urinary tract urothelial carcinoma; AA, aristolochic acid; BMI, body mass index; NLR, neutrophil-lymphocyte ratio.

**Supplementary Table 6** Univariate and multivariate analyses for factors associated with BC with at least one unfavorable pathological type after RNU.

|  | **Operation interval** $\boldsymbol{\leq}$ **1 year** | | | | **Operation interval** $\boldsymbol{>}$ **1 year** | | | |
| --- | --- | --- | --- | --- | --- | --- | --- | --- |
|  | **Univariate analysis** | | **Multivariate analysis** | | **Univariate analysis** | | **Multivariate analysis** | |
|  | OR (95% CI) | P | OR (95% CI) | P | OR (95% CI) | P | OR (95% CI) | P |
| Operation Interval | 1.085(0.902-1.304) | 0.386 |  |  | 1.015(0.987-1.044) | 0.287 |  |  |
| Stage of UTUC ($<$T2 vs. $\geq$T2) | 1.222(0.439-3.401) | 0.701 |  |  | 0.652(0.181-2.350) | 0.513 |  |  |
| AA |  |  |  |  | 0.943(0.103-8.598) | 0.958 |  |  |
| Age | 0.966(0.917-1.018) | 0.202 |  |  | 1.037(0.981-1.097) | 0.199 |  |  |
| BMI | 0.940(0.805-1.098) | 0.435 |  |  | 1.125(0.931-1.359) | 0.222 |  |  |
| BMI ($<$25 vs. $\geq$25) | 1.582(0.541-4.624) | 0.402 |  |  | 2.067(0.518-8.251) | 0.304 |  |  |
| Diameter of UTUC  ($\leq$ 3cm vs. $>$ 3cm) | 0.726(0.254-2.076) | 0.550 |  |  | 1.378(0.381-4.978) | 0.625 |  |  |
| Drinking | 0.475(0.107-2.107) | 0.327 |  |  | 0.611(0.062-5.983) | 0.672 |  |  |
| Gender | 1.206(0.438-3.320) | 0.717 |  |  | 0.643(0.187-2.206) | 0.482 |  |  |
| Grade of UTUC | 1.533(0.532-4.419) | 0.429 |  |  | 1.753(0.502-6.120) | 0.379 |  |  |
| Hydronephrosis | 0.846(0.303-2.365) | 0.750 |  |  | 1.095(0.318-3.771) | 0.885 |  |  |
| Multifocal tumor | 0.833(0.259-2.679) | 0.760 |  |  | 0.677(0.162-2.830) | 0.593 |  |  |
| Surgical approach | 0.184(0.038-0.893) | 0.036 |  |  | 0.753(0.219-2.583) | 0.652 |  |  |
| NLR | 0.917(0.722-1.163) | 0.474 |  |  | 1.073(0.863-1.333) | 0.526 |  |  |
| NLR ($<$2.5 vs. $\geq$2.5) | 0.536(0.192-1.495) | 0.233 |  |  | 0.556(0.154-2.002) | 0.369 |  |  |
| Smoking | 0.944(0.298-2.995) | 0.923 |  |  | 0.738(0.178-3.063) | 0.675 |  |  |
| Ureteroscopy | 0.729(0.223-2.381) | 0.600 |  |  |  |  |  |  |
| Location |  |  |  |  |  | 0.913 |  |  |
| (ureter vs. renal pelvis) |  |  |  |  | 1.042(0.291-3.726) | 0.950 |  |  |
| (both vs. renal pelvis) |  |  |  |  | 0.625(0.058-6.790) | 0.699 |  |  |

**Abbreviations:** BC, bladder cancer; RNU, Radical nephroureterectomy; OR, odds ratio; CI=confidence interval; UTUC, upper urinary tract urothelial carcinoma; AA, aristolochic acid; BMI, body mass index; NLR, neutrophil-lymphocyte ratio.
